# Risk Factors Associated with Children Missing the Fourth Dose of DTaP Vaccination

**DOI:** 10.9734/BJMMR/2015/16117

**Published:** 2015-02-10

**Authors:** Zhen Zhao

**Affiliations:** 1 National Center for Immunization and Respiratory Diseases, Centers for Disease Control and Prevention, 1600 Clifton Road NE, Mail Stop A19, Atlanta, GA 30333, USA.

**Keywords:** Vaccines, parents, providers, timeliness, confidences, pertussis

## Abstract

**Background:**

In 2012, reported pertussis reached the highest number of cases (48,277) in the United States since 1955.

**Objectives:**

Estimate the prevalence of children who missed the fourth dose of DTaP (Diphtheria and Tetanus toxoids and acellular Pertussis vaccine) by parents’ confidences in vaccines and influences from providers, the timeliness of the first through the third dose of DTaP, and selected socio-demographic characteristics; identify the significant risk factors for non-receipt of the fourth dose of DTaP; and evaluate the unadjusted and adjusted risk ratios for missing the fourth dose of DTaP.

**Methods:**

Data from 16,919 children 19–35 months living in the United States included in the 2011 National Immunization Survey were analyzed. Weighted categorical data analysis and multivariable regression in the context of complex sample survey were applied to assess the prevalence and to determine the independent risk factors.

**Results:**

Overall, 14.7% of children missed the fourth dose of DTaP. Children who were late in receiving the third dose of DTaP had significantly higher risk of missing the fourth dose of DTaP than children who were on-time in receiving the third dose of DTaP (adjusted risk ratio (RR) 2.48; 95%CI (1.92, 3.20)). The risk of missing the fourth dose of DTaP was 62% higher among children whose parents reported they didn’t have a good relationship with their child’s health-care providers than children whose parents reported having good relationship. Compared with the risk of missing the fourth dose of DTaP among children whose parents were confident in the value of vaccines, the risk was significantly higher for the children whose parents lacked confidence (adjusted RR 1.41; 95%CI (1.05, 1.89)).

**Conclusions:**

Timeliness in receiving the first through the third dose of DTaP, influences from providers, and parents’ confidence in the value of vaccines are the five significant risk factors for missing the fourth dose of DTaP vaccination. They are all modifiable. Future interventions to improve parental relationships with providers and attitudes toward vaccines could help improve pertussis vaccination coverage.

## 1. INTRODUCTION

Pertussis, or whooping cough, is a highly contagious infectious disease caused by the bacterium *Bordetella pertussis*. In 2012, reported pertussis reached the highest number of cases (48,277) in the United States since 1955; and 2.6 times the cases reported in 2011. Eighteen children died. In addition, the reported pertussis incidence reached 126.7 per hundred thousand among infants < 1 year, and 34.1 for children 1–6 years old, the highest since 1990 [1]. Pertussis can be prevented with vaccines. In the United States, four doses of Diphtheria and Tetanus toxoids and acellular Pertussis vaccine (DTaP) are routinely recommended in early childhood, by the Advisory Committee on Immunization Practices (ACIP), the American Academy of Pediatrics (AAP), and the American Academy of Family Physicians (AAFP) [2]. The necessity for four doses of DTaP is based on the continual reoccurrence or outbreaks of pertussis among young children in the United States, and sustaining adequate immunity during preschool years [3–4]. Without proper DTaP vaccination, infants are at risk for getting pertussis and then having severe complications from it, including death. About half of infants younger than 1 year old who get pertussis are hospitalized, and 1 or 2 in 100 hospitalized infants die [5]. The fourth dose of DTaP is critical in boosting antibody titer and insuring continuous protection [6]. Several studies have indicated that the fourth dose of DTaP is among the commonly missed vaccines for children who are not adequately immunized [7–13]. In 2011, an estimated 14.7% (approximately 1 million) of children in the United States were at risk because they had not received their fourth dose of DTaP [14].

The associations of parents’ confidences in vaccines and influences from providers with children missing the fourth dose of DTaP have not been fully investigated. Therefore, this observational study has been conducted using a national representative sample of 16,919 children aged 19–35 months in the United States obtained from the 2011 National Immunization Survey. This study estimated the prevalence of children who missed the fourth dose of DTaP by parents’ confidences in vaccines and influences from providers, child’s health insurance status, the timeliness of the first through the third dose of DTaP vaccination, and selected sociodemographic characteristics; identified the significant risk factors for non-receipt of the fourth dose of DTaP; and evaluated the unadjusted and adjusted risk ratios for missing the fourth dose of DTaP for significant risk factors.

## 2. METHODS

### 2.1 Data Resources

The National Immunization Survey (NIS) landline sample data collected from 2011 was used for this study. The NIS is conducted annually by the Centers for Disease Control and Prevention (CDC) to obtain national, state, and selected local area estimates of vaccination coverage for the U.S. non-institutionalized population of children 19–35 months [15]. The NIS is a random-digit-dialed telephone survey of households with age eligible children followed by a mail survey to sample children’s providers to obtain provider-reported vaccination histories. In the 2011 NIS, the overall landline telephone response rates based on Council of American Survey and Research Organizations (CASRO) guidelines was 61.5%. Immunization information and parental attitudes toward vaccines obtained for the 16,919 children of 19–35 months living in the United States were analyzed. Smith et al. [16] provide a detailed description of the statistical methods used by the NIS, which has been approved by CDC Institutional Review Board.

### 2.2 Vaccination Status, Child, Maternal and Family Factors

According to the ACIP vaccination recommendation, the children were on time for the first through the third dose of DTaP, if they received the doses by 2, 4, and 6 months, respectively (i.e., before turning 3, 5, and 7 months, respectively), otherwise the children were late for the first through the third dose of DTaP [13]. Dose 4 was considered on time if it was administered between 15 to 18 months of age (The fourth dose may be administered as early as 12 months, provided at least 6 months have elapsed since the third dose). Dose 4 was considered late if it was received at or after 19 months. The 4 doses of DTaP are recommended for all children since 1991 by ACIP [17–18].

Children were defined as having health insurance if they were covered through the parents’ employer or union; Medicaid; S-CHIP; Indian Health Service; Military Health Care, Tricare, Champus, or Champ-VA; or Other Health Insurance or Health Care Plan.

The socio-demographic factors related to child, maternal, family, and vaccination providers available in the NIS were examined in this study. Those factors had previously been found to be associated with childhood vaccination coverage in the United States [19–21]: child’s first born status (yes vs. no), and number of siblings (0 vs. ≥1); family locality (urban, suburban, rural), and mobility status since birth (not moved vs. moved); mother’s education level (≤12 years vs. ≥ 13 years), mother’s marital status (married vs. not married), and age group of mother (≤29 years vs. ≥30 years); number of child’s vaccination providers (1 vs. ≥2) and type of providers (public, other, private).

### 2.3 Assessment of Parents’ Confidences in Vaccines and Influences from Providers

In order to efficiently and effectively predict parents’ decisions to vaccinate their children with recommended vaccines, La Vail and Kennedy [22] proposed four measures of parental attitudes toward vaccines: parents’ confidence in the value of vaccines, parents’ confidence in the efficacy of vaccines, parents’ confidence in the safety of vaccines, and influences from providers; the four measures index the parents’ beliefs about vaccines. In this study, those four measures were applied to investigate the associations of parents’ confidences in vaccines and influences from providers with children missing the fourth dose of DTaP vaccination. From the 2011 NIS data, four statements read to parents and the parents’ responses were selected to best represent the four measures. The four statements are how much the parent or guardian agrees/disagrees with: (1) “vaccines are necessary to protect the health of children”; (2) “if I do not vaccinate my child, he/she may get a disease such as measles and cause other children or adults also to get the disease”; (3) “vaccines are safe”; and (4) “I have a good relationship with my child’s health-care provider”. The parent or guardian provides a verbatim response on a scale of zero to 10, where zero meant strongly disagree, and 10 meant strongly agree. In this study, it is assumed that a verbatim response of ≥7 were more likely to agree with the statements than those who gave a response of ≤6. For convenience, if parents provided a verbatim response of ≥7, the parents are believed to agree with the statement [23], otherwise ≤6 was categorized as parents disagree. Thus corresponding to the four statements selected above, the four new variables are created: (1) parents’ confidence in the value of vaccines (no vs. yes); (2) parents’ confidence in the efficacy of vaccines (no vs. yes); (3) parents’ confidence in the safety of vaccines (no vs. yes); and (4) influences from providers (no vs. yes). To evaluate the association of parents’ confidence in the value, efficacy, safety of vaccines, and influence from providers with children missing the fourth dose of DTaP was the major objective of this study.

### 2.4 Statistical Methods

All of the analyses in this study were performed using SUDAAN 11.0.0 [24], which properly accounts for the complex sampling survey design in the NIS. The prevalence rate for missing the fourth dose of DTaP vaccination was estimated using weighted categorical data analysis; weighted prevalence rates with 95% confidence interval (CI) were obtained; P-value of the Chisquare test was used to evaluate the association of each factor with missing the fourth dose of DTaP vaccination. Backward stepwise multivariable logistic regression [25] was conducted to obtain the final model, which comprises the significant and independent factors for missing the fourth dose of DTaP vaccination. Multicollinearity was assessed for all factors in the final multivariable logistic regression model [26]. Unadjusted and adjusted risk ratios (risk of missing the fourth dose of DTaP vaccination in one category of the factor compared to the risk in the reference category for the same factor) were estimated for each of the factors in the final model.

## 3. RESULTS

### 3.1 Prevalence of Missing the Fourth Dose of DTaP vaccination

**Fig. 1.**
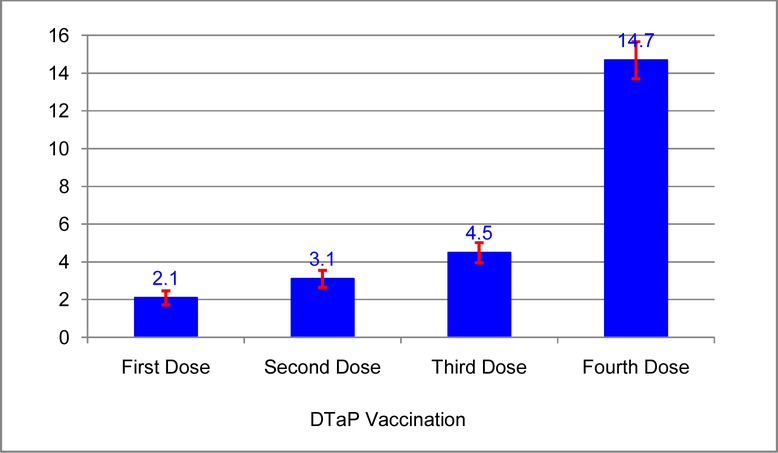
Weighted percentage (%) and 95%CI^a^ of children missing the first, second, third, and fourth dose of DTaP^b^ vaccination, National Immunization Survey 2011 ^a^95% Confidence Interval ^b^Diphtheria and Tetanus toxoids and acellular Pertussis

In 2011, among children aged 19–35 months in the United States, an estimated 14.7% (approximately 1 million) missed their fourth dose of DTaP vaccination. About 2.1% (125 thousands) of children missed the first dose of DTaP (i.e., missed all doses of DTaP vaccination) (Fig. 1). Prevalence rates and 95% confidence intervals for missing the fourth dose of DTaP vaccination by parents’ confidences in vaccines, influences from providers, timeliness of the first through the third dose of DTaP, health insurance status of children, and selected socio-demographic characteristics are presented in Table 1. The prevalence rates ranged from 6.2% to 41.9%. Among the three parents’ vaccine confidence factors, children whose parents lacked confidence in the value of vaccines had the highest prevalence rate, indicating about 39.9% missed the fourth dose of DTaP vaccination. Approximately 33.1% of children missed the fourth dose of DTaP vaccination if their parents didn’t have a good relationship with their healthcare provider. The three “Late” categories for Timeliness of the first through the third dose of DTaP had the highest group prevalence rates of 41.9%, 34.5%, and 26.7% for missing the fourth dose of DTaP respectively. Among children who did not have health insurance, 21.0% missed the fourth dose of DTaP vaccination. Children whose mother’s age were 29 years or younger had significantly higher prevalence rate of 19.7% compared to the prevalence rate of 12.9% among children whose mother were 30 years or older. About 17.4% of children missed the fourth dose of DTaP vaccination if they were not first born. The difference in prevalence rates among categories of each factor was significant (P-value< 0.01) for all 13 factors.

### 3.2 Risk Factors and Risk Ratios for Missing the Fourth Dose of DTaP Vaccination

Seven significant and independent risk factors for missing the fourth dose of DTaP were identified and are included in Table 2. They include parents’ confidence in the value of vaccines, parents have a good relationship with providers, timeliness of the first through the third dose of DTaP, mother’s age group, and child’s first born status. Children who were late in receiving the third dose of DTaP vaccination had significantly higher risk of missing the fourth dose of DTaP vaccination than the risk for children who were on-time in receiving the third dose of DTaP vaccination (adjusted risk ratio (RR) 2.48; 95%CI (1.92, 3.20)). The risk of missing the fourth dose of DTaP was 62% higher (adjusted RR 1.62; 95%CI (1.10, 2.38)) among children whose parents didn’t have a good relationship with their child’s health-care providers than children whose parents did have a good relationship. Comparing with the risk of missing the fourth dose of DTaP vaccination among children whose parents were confident in the value of vaccines, the risk was significantly higher for the children whose parents lacked confidence in the value of vaccines (adjusted RR 1.41; 95%CI (1.05, 1.89)). In addition, the late receipt of the first and second dose of DTaP vaccination was associated with a significantly higher risk for non-receipt of the fourth dose of DTaP vaccination. Other factors significantly associated with missing the fourth dose of DTaP vaccination were sociodemographic characteristics, including children whose mother was 29 years or younger (adjusted RR 1.40; 95% CI (1.16, 1.69)), and children who were not the first born child in their family (adjusted RR 1.26; 95% CI (1.03, 1.54)).

## 4. DISCUSSION

Children who missed the fourth dose of DTaP not only leave the children susceptible to pertussis, but also make their communities vulnerable to outbreaks of pertussis. Why children missed the fourth dose of DTaP vaccination? Results from this study suggest that the major problems depend on the timeliness in receiving vaccination, influences from providers, and parents’ confidences in vaccines. For example, this study found that the main reasons for missing the fourth dose of DTaP are among the children who received the first, second, and third dose of DTaP late. These findings are similar to Strine’s work [13] which evaluated the predictors of age-appropriate receipt of the fourth dose of DTaP. But, Strine’s study did not identify that the timeliness of the first dose of DTaP vaccination was a significant factor for receiving the fourth dose of DTaP vaccination; in addition their study did not consider the factors of parents’ confidences in vaccines and parents’ relationship with health care providers, which are the major drawback of their article. However, this current study showed that children missing the fourth dose of DTaP is significantly associated with the influences from their healthcare providers and parents’ confidence in the value of vaccines, these findings are concordant with other researches that demonstrated parental attitudes toward vaccination were significant predictor for the vaccination coverage of children [23,27]; nevertheless those two studies did not control the timeliness of the first through the third dose of DTaP vaccination in their analyses. It is encouraging that this current study incorporates parents’ confidences in vaccines, timeliness of the first through the third dose of DTaP vaccination, health insurance status of children, and available important socio-demographic factors in NIS, and evaluated the potential risk factors for missing the fourth dose of DTaP. To our knowledge, this article is a comprehensive and in-depth study which identified and demonstrated the five significant and modifiable risk factors that will be helpful to health care professional in their efforts to control and prevent pertussis.

Best-designed and carefully implemented communication between parents and health care providers can establish parents’ confidence in the value of vaccines and create good relationship of parents with health care providers. Parents want to trust and receive immunization information from their child’s providers [28–33] in making decisions about their children’s vaccinations. Therefore, health care providers are very important for building parents confidence in the value, efficacy, and safety of vaccines and for providing the guidance and advice about vaccinating their children. It is critical that providers be able to devote the time and effort needed to communicate immunization information effectively to parents. Understandably, providers need to keep the child’s visits brief, especially in busy medical practices; however, there is a need to engage in more in-depth communication, interactions, and collaborations with parents [34]. Marvel and colleagues [35] found that family physicians with training in family therapy methods—particularly in communication and counseling skills—did not have longer patient visits but nonetheless engaged in more in-depth interactions and collaborations with patients than did physicians without such training. Rosen stock et al. [36] also suggested that parents who are more vaccine-hesitant are likely to be influenced only through personal, face-to-face contact, especially with their physician. The American Academy of Pediatrics has published guidelines for physicians on how to engage parents and get them to talk about their concerns about vaccines [37]. By using the authority that parents customarily confer upon traditional health-care providers in a respectful, non-coercive, and noncondescending manner, and by using logicsupported scientific knowledge about vaccines, traditional health-care providers who listen to the concerns of parents with an empathetic ear [38] will be in the best position to lead vaccine hesitant parents to make their own informed decision that vaccinating their children is the best way to protect their children from vaccine preventable diseases. To effectively communicate with vaccine-hesitant parents, health care providers (HCPs) must first understand the concerns of parents regarding immunization and understand influences that can lead to misinformation about vaccines. HCPs should establish an open, non-confrontational dialogue with parents at an early stage and provide unambiguous, easily comprehensible answers about known vaccine adverse events and provide accurate information about vaccination. Ongoing dialogue including provider recommendations may successfully reassure vaccine-hesitant parents that immunization is the best and safest option for their child [39]. It is essential for public health organizations and medical societies to continue their efforts to assist or augment physicians’ efforts to effectively communicate with parents about immunizations. For example, the CDC recently developed and distributed a provider resource kit designed to effectively and efficiently provide immunization information to parents in providers’ offices [40].

Client Reminder and Recall are effective intervention that can help increase receipt of timely DTaP vaccination [41]. In order to increase appropriate vaccination including the fourth dose of DTaP, CDC’s Guide to Community Preventive Services Task Force recommends Client Reminder and Recall Intervention System: Client reminder and recall interventions involve reminding members of a target population that vaccinations are due (reminders) or late (recall). Reminders and recalls differ in content and are delivered by various methods—telephone, letter, postcard, or other. Most reminder systems involve a specific notification for a specific client, and may be accompanied by educational messages regarding the importance of immunization for the targeted vaccine. Client reminder and recall interventions are recommended based on strong evidence of effectiveness in improving vaccination coverage. The previous review (search period 1980–1997) included 54 study arms from 42 studies with a median absolute increase in vaccination coverage of 12.0 percentage points. Thirty-four study arms evaluated client reminder and recall when implemented alone (median absolute increase in vaccination coverage of 8.0 percentage points), and nine studies examined this intervention with additional components (median absolute increase in vaccination coverage of 16.0 percentage points). The updated review identified 20 additional studies from 19 papers (search period 1997–2007) with a median absolute increase in vaccination coverage of 6.1 percentage points. Twelve studies examined the impact of client reminder and recall alone and documented a median absolute increase of 5.1 percentage points. Eight studies evaluated client reminder and recall interventions with additional components, and documented a median absolute increase of 11.0 percentage points. The reviewed studies evaluated the effectiveness of client reminder and recall in a wide range of client and provider populations and settings. No evidence of harms regarding the use of client reminder and recall was identified in either the 1997 review or in the 2007 update.

One of the potential limitations of this study is that this is an observational study, and it cannot demonstrate a causal relationship between factors and missing the fourth dose of DTaP vaccination. In addition, this study used the 2011 NIS data from the landline telephone sample and did not account for children who live in households with cellular phone service only. Therefore, the estimates presented in this study could be biased since households not covered by the landline telephone sample might be different from those that are covered by the landline sample with respect to the outcomes that we have reported. However, recent work suggests that bias in surveys that only sample households with landline telephones maybe small [42–43].

## 5. CONCLUSIONS

In view of the resurgence of pertussis disease during 2012 in the United States [44], the fourth dose of DTaP is critical in protecting children from pertussis infection, and approximate one million of children in the United States missed the fourth dose of DTaP vaccination, that there is a need to know how and why the children missed the fourth dose of DTaP. To address those two questions, this current study estimated the prevalence of children who missed the fourth dose of DTaP vaccination by parents’ confidences in vaccines and influences from providers, the timeliness of the first through the third dose of DTaP, and selected sociodemographic characteristics; identified the significant risk factors for missing the fourth dose of DTaP; and evaluated the unadjusted and adjusted risk ratios for missing the fourth dose of DTaP controlling the covariates. This study found that timeliness in receiving the first through the third dose of DTaP, influences from providers, and parents’ confidence in the value of vaccines are significant and modifiable risk factors [45–46] for missing the fourth dose of DTaP vaccination. Future interventions focusing on those modifiable risk factors could help to increase DTaP vaccination and reduce pertussis infection among children in the United States.

## Figures and Tables

**Table 1.** Prevalence for missing the fourth dose of DTaP^a^ vaccination by parents’ confidences in vaccines and influences from providers, timeliness^b^ of the first through the third dose of DTaP, health insurance status of children, and selected socio-demographic factors, 2011 National Immunization Survey

Factors		Un-weighted sample size (weighted %)	Weighted prevalence of missing the fourth dose of DTaP and significant level	
			% (95% CI^c^)	Chi-Square P-value
National		16919	14.7 (13.7, 15.7)	
Confidence in the Value of Vaccines^d^	No	713 (5.8)	39.9 (34.1,46.0)	< 0.01
	Yes	11190 (94.2)	14.1 (13.0, 15.3)	
Confidence in the Efficacy of Vaccines^e^	No	1685 (16.1)	21.8 (18.4,25.7)	< 0.01
	Yes	10132 (83.9)	14.3 (13.1, 15.5)	
Confidence in the Safety of Vaccines^f^	No	2150 (18.8)	23.9 (20.8, 27.2)	< 0.01
	Yes	9692 (81.2)	13.8 (12.6, 15.0)	
Parents have good relationship with Providers^g^	No	548 (5.8)	33.1 (26.0,41.0)	< 0.01
	Yes	11310 (94.2)	14.4 (13.4, 15.6)	
Timeliness of the First dose of DTaP	Late	1431 (10.3)	41.9 (37.3,46.6)	< 0.01
	On-time	15116 (89.7)	10.4 (9.6, 11.3)	
Timeliness of the Second dose of DTaP	Late	2581 (18.2)	34.5 (31.4,37.8)	< 0.01
	On-time	13831 (81.8)	7.9 (7.1,8.8)	
Timeliness of the Third dose of DTaP	Late	3879 (26.4)	26.7 (24.3, 29.2)	< 0.01
	On-time	12321 (73.6)	6.2 (5.4, 7.0)	
Health insurance status of child	No	2448 (18.7)	21.0 (18.6,23.5)	< 0.01
	Yes	14471 (81.3)	14.2 (13.2, 15.3)	
Education level of Mothers	≤ 12 years	4538 (47.8)	18.5 (16.8,20.2)	< 0.01
	≥ 13 years	12381 (52.2)	12.7 (11.7, 13.8)	
Age group of mothers	≤ 29 years	5201 (38.0)	19.7 (17.9,21.7)	< 0.01
	≥ 30 years	11718 (62.0)	12.9 (11.8, 14.0)	
Type of vaccination providers	Public	1727 (13.0)	20.3 (17.2,23.7)	< 0.01
	Other	4893 (27.8)	17.8 (15.8,20.0)	
	Private	10136 (59.2)	12.2 (11.2, 13.4)	
Family mobility	Moved	1255 (6.6)	23.7 (19.6,28.5)	< 0.01
	Not moved	15664 (93.4)	14.9 (13.9, 15.9)	
First born child	Not	11261 (67.1)	17.4 (16.2, 18.8)	< 0.01
	Yes	5658 (32.9)	11.5 (10.1, 13.0)	

a Diphtheria and Tetanus toxoids and cellular Pertussis vaccine

b Timeliness are defined for the first, second, third, and fourth dose of DTaP vaccination for all children as On-time, Late, and Missing respectively

c Confidence Interval

d Measure of Parents’ Confidence in the Value of Vaccines is evaluated by parents response to the question “Vaccines are necessary to protect the health of children”, the response score ranged from 0 to 10. If the response score is ≥ 7, the confidence in the value of vaccines is defined as “Yes”, otherwise the confidence is defined as “No”

e Measure of Parents’ Confidence in the Efficacy of Vaccines is evaluated by parents response to the question “If I do not vaccinate my child, he/she may get a disease and cause other children or adults also to get the disease”, the response score ranged from 0 to 10. If the response score is ≥ 7, the confidence in the efficacy of vaccines is defined as “Yes”, otherwise the confidence is defined as “No”.

f Measure of Parents’ Confidence in the Safety of Vaccines is evaluated by parents response to the question “Vaccines are safe”, the response score ranged from 0 to 10. If the response score is ≥ 7, the confidence in the safety of vaccines is defined as “Yes”, otherwise the confidence is defined as “No”.

g Measure of the Influences from Providers is evaluated by parents response to the question “I have a good relationship with my child’s health-care provider”, the response score ranged from 0 to 10. If the response score is ≥ 7, parents have good relationship with providers is defined as “Yes”, otherwise the relationship is defined as “No”

**Table 2.** Unadjusted and adjusted risk ratios for missing the fourth dose of DTaP^a^ by the significant factors obtained from the final multivariable logistic model among children 19–35 months in the United States, 2011 National Immunization Survey

Factors	Comparison	Unadjusted risk ratios		Adjusted risk ratios	
		% (95%CI^b^)	P-value (Wald ChiSq)	% (95%CI^b^)	P-value (Wald ChiSq)
Confidence in the Value of Vaccines^c^	No vs. Yes	2.82 (2.38, 3.34)	< 0.01	1.41 (1.05, 1.89)	< 0.03
Parents have good relationship with providers^d^	No vs. Yes	2.29 (1.80,2.91)	< 0.01	1.62 (1.10,2.38)	< 0.02
Timeliness of the First dose of DTaP	Late vs. On-time	4.02 (3.50, 4.62)	< 0.01	1.42 (1.09, 1.86)	< 0.01
Timeliness of the Second dose of DTaP	Late vs. On-time	4.38 (3.80, 5.04)	< 0.01	1.52 (1.15,2.00)	< 0.01
Timeliness of the Third dose of DTaP	Late vs. On-time	4.32 (3.69, 5.06)	< 0.01	2.48 (1.92,3.20)	< 0.01
Age group of mothers	≤ 29 vs. ≥ 30	1.53 (1.35, 1.74)	< 0.01	1.40 (1.16, 1.69)	< 0.01
First born child	Not vs. Yes	1.52 (1.32, 1.76)	< 0.01	1.26 (1.03, 1.54)	<0.03

a Diphtheria and Tetanus toxoids and cellular Pertussis;

b Confidence Interval

c Measure of Parents’ Confidence in the Value of Vaccines is evaluated by parents response to the question “Vaccines are necessary to protect the health of children”, the response score ranged from 0 to 10. If the response score is ≥ 7, the confidence in the value of vaccines is defined as “Yes”, otherwise the confidence is defined as “No”.

d Measure of the Influences from Providers is evaluated by parents response to the question “I have a good relationship with my child’s health-care provider”, the response score ranged from 0 to 10. If the response score is ≥ 7, parents have good relationship with providers is defined as “Yes”, otherwise the relationship is defined as “No”
